# The New Frontier of JAK Inhibitors: Significant Therapeutic Response to Tofacitinib in a Patient With Granulomatous Reaction to Filler in the Buttocks

**DOI:** 10.1111/jocd.16578

**Published:** 2024-10-15

**Authors:** Parvin Mansouri, Susan Farshi

**Affiliations:** ^1^ Skin and Stem Cell Research Center Tehran University of Medical Sciences Tehran Iran

**Keywords:** granuloma, JAK inhibitors, tofacitinib

## Abstract

**Background:**

Recent research has demonstrated that Janus Kinase (JAK) inhibitors can be effective in treating refractory granulomatous diseases.

**Case Presentation:**

We report the case of a 50‐year‐old woman who developed a granulomatous reaction following a filler injection in her buttocks.

**Management:**

The patient was treated with tofacitinib, and after 1 year of therapy, the stiffness and swelling resolved without any side effects.

**Conclusion:**

Tofacitinib appears to be a viable option for the treatment of granulomatous reactions to fillers.

## 1 Case Presentation

A 50‐year‐old woman presented to our clinic with bilateral sclerotic, hyperpigmented lesions in the buttocks. She had received a filler injection, likely containing oils, in her buttocks 1 year prior. Shortly after the injection, she experienced swelling, stiffness, and severe pain in the area. Although her doctor attempted to drain the filler, only a portion was removed, and the symptoms persisted. She was treated with oral prednisolone (30 mg/day) and cyclosporine (3 mg/kg/day) for several months, but without improvement. Intralesional triamcinolone worsened her condition. A skin biopsy revealed extensive dermal collagen necrobiosis with palisaded granulomatous lymphohistioplasmacytic infiltrate and a low‐grade vasculopathic reaction, suggestive of necrobiosis lipoidica. No acid‐fast bacilli or fungal elements were detected in special stains.

During our examination, we observed infectious secretions from the affected area and initiated treatment with clindamycin (300 mg TDS), rifampin (300 mg BD), and linezolid (600 mg BD). We also drained the secretions. A smear and culture performed prior to antibiotic therapy revealed a mixed bacterial infection.

Routine laboratory tests, including CBC, ESR, BUN, Cr, uric acid, triglycerides, cholesterol (HDL and LDL), TFT (T3, T4, TSH, and T3RU), LFT (AST, ALT, and alkaline phosphatase, bilirubin), HBsAg, HBsAb, HbcAb, HCVAb, HIV, PPD or IGRA, and chest X‐ray, all returned normal results. An ultrasound revealed multiple hypoechoic regions in both buttocks, consistent with fluid collections. Although an MRI had been performed before the patient presented to us, she neither had the report nor was willing to undergo another MRI.

Standardized photographs were taken before the treatment began, and follow‐up photographs were taken in the same positions during subsequent visits.

Given the severity of her skin stiffness, which interfered with her daily activities, and considering the anti‐fibrotic effects of methotrexate, we initiated oral methotrexate at 15 mg/week. After 2 months without significant improvement, we started tofacitinib at 5 mg BD. Three months later, the buttock area had softened slightly, the pain had subsided, and there was no longer any pus drainage. Encouraged by this positive initial response and the absence of adverse effects, we increased the tofacitinib dose to 10 mg BD after 6 months. Due to the patient's complaint of hyperpigmentation, we also performed intense pulse light (IPL) therapy and injected tranexamic acid (50 mg/mL) intradermally into the hyperpigmented areas. She underwent six monthly sessions of IPL and tranexamic acid injections.

**FIGURE 1:**
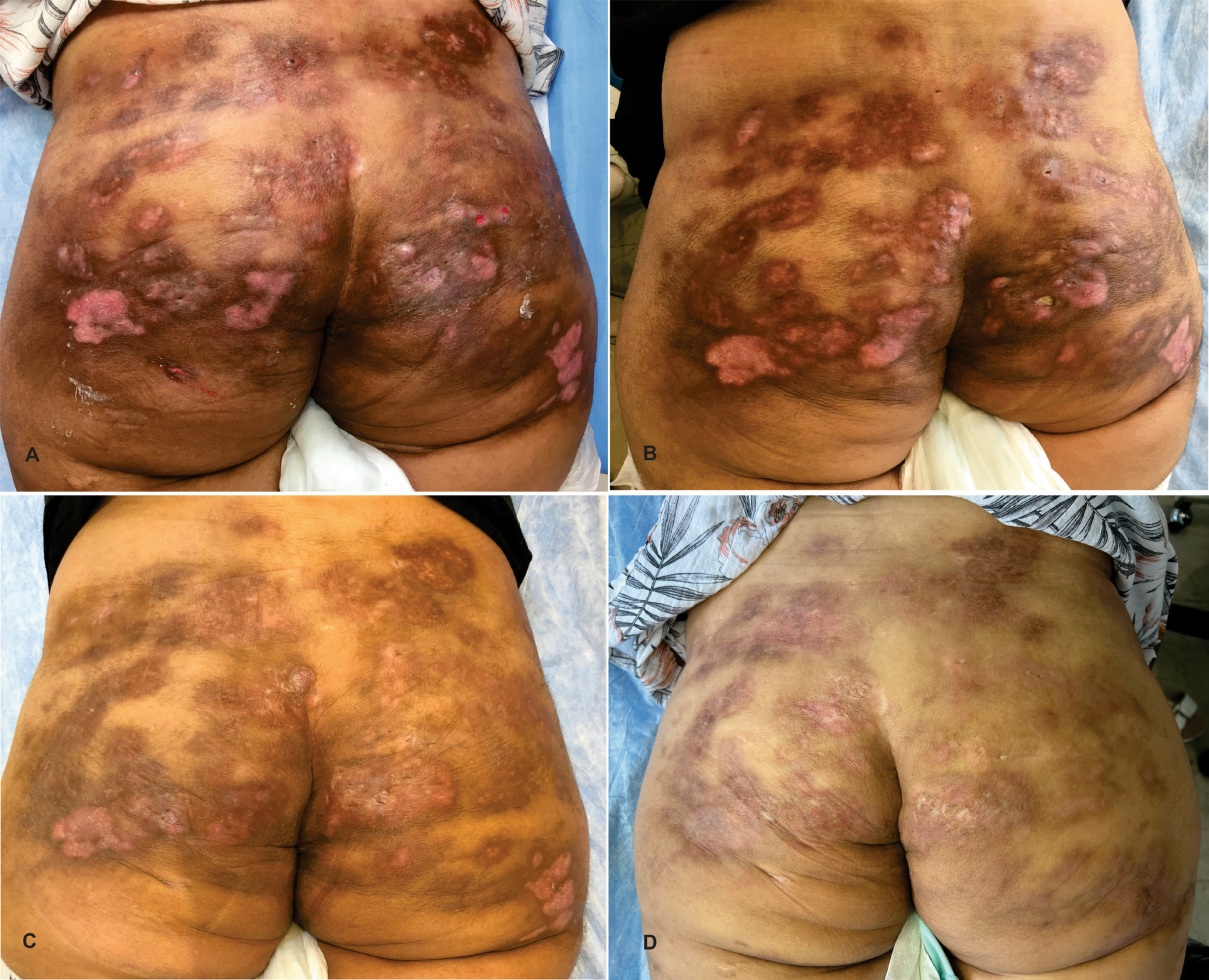
Patient's buttocks before treatment (A), after 2 months (B), after 6 months (C), and after 1 year of tofacitinib therapy (D).

Figure 1 shows the patient's buttocks before treatment (A), after 2 months (B), after 6 months (C), and after 1 year of tofacitinib therapy (D). No adverse events were reported during the year‐long use of tofacitinib, and the treatment was well tolerated.

## 2 Discussion

Granulomatous inflammation and granulomas are common reactions to foreign bodies [1]. Granuloma foreign body reaction is a type of delayed hypersensitivity reaction mediated by T cells and is one of the complications of dermal fillers [2]. Filler removal through manual aspiration is rarely successful, especially in granulomatous and infected lesions, often necessitating surgical excision. Macrophage activation and accumulation in tissue lead to the formation of granulomas. Some studies have shown that overproduction of inflammatory cytokines and subsequent activation of the JAK–STAT pathway may have a role in granulomatous disorders. Consequently, the use of JAK inhibitors, which block this pathway, has demonstrated improvements in sarcoidosis and granuloma annulare patients [1, 3].

A study has shown that JAK inhibition might be an effective treatment option in refractory necrobiosis lipoidica. This study showed that JAK inhibition alone normalized wound healing [4]. JAK inhibitors have also shown potential in inducing remission in sclerodermatous and fibrotic skin diseases. The beneficial effect of tofacitinib in other skin autoimmune diseases has been recently demonstrated [5]. Interleukin (IL)‐4 and transforming growth factor‐β (TGF‐β) play significant roles in morphea pathogenesis by promoting collagen and extracellular matrix overproduction by fibroblasts, processes that are inhibited by JAK blockade [6]. Improvement in skin thickness has been observed in a patient with generalized morphea treated with tofacitinib 5 mg BD for 16 months [7].

Our case demonstrated satisfactory results with tofacitinib in treating a granulomatous reaction to filler, though further controlled studies are needed to assess the long‐term effects and safety. This case report may pave the way for new indications of JAK inhibitors in the management of granulomatous reactions to fillers, offering a new approach to this challenging complication.

## Ethics Statement

The study protocol conformed to the guidelines of the 1975 Declaration of Helsinki.

## Conflicts of Interest

The authors declare no conflicts of interest.

## Data Availability

The data that support the findings of this study are available from the corresponding author upon reasonable request.
